# Complex environmental drivers of immunity and resistance in malaria mosquitoes

**DOI:** 10.1098/rspb.2013.2030

**Published:** 2013-11-07

**Authors:** Courtney C. Murdock, Lillian L. Moller-Jacobs, Matthew B. Thomas

**Affiliations:** Center for Infectious Disease Dynamics, Department of Entomology, Pennsylvania State University, Merkle Lab, Orchard Road, University Park, PA 16802, USA

**Keywords:** innate immunity, mosquito, temperature, circadian rhythm, resistance

## Abstract

Considerable research effort has been directed at understanding the genetic and molecular basis of mosquito innate immune mechanisms. Whether environmental factors interact with these mechanisms to shape overall resistance remains largely unexplored. Here, we examine how changes in mean ambient temperature, diurnal temperature fluctuation and time of day of infection affected the immunity and resistance of *Anopheles stephensi* to infection with *Escherichia coli*. We used quantitative PCR to estimate the gene expression of three immune genes in response to challenge with heat-killed *E. coli*. We also infected mosquitoes with live *E. coli* and ran bacterial growth assays to quantify host resistance. Both mosquito immune parameters and resistance were directly affected by mean temperature, diurnal temperature fluctuation and time of day of infection. Furthermore, there was a suite of complex two- and three-way interactions yielding idiosyncratic phenotypic variation under different environmental conditions. The results demonstrate mosquito immunity and resistance to be strongly influenced by a complex interplay of environmental variables, challenging the interpretation of the very many mosquito immune studies conducted under standard laboratory conditions.

## 1. Introduction

Throughout the past two decades, researchers have made great strides in describing the innate immune system of mosquito vectors [1], in defining the key genetic players shaping mosquito resistance to vector-borne pathogens (such as malaria and dengue virus) [2–4] and in identifying potential targets for genetic manipulation [5–7]. By and large, most of this research has been conducted under simplified laboratory conditions with tight control over variables, like environmental temperature. Yet, mosquitoes and their parasites associate in a variable environment [8–10]. Studies on a wide range of invertebrates show that small, realistic changes in ambient temperature can shape host resistance (reviewed in [11]). Further, it is well established that temperature has diverse impacts on mosquito physiology [12–14] and the development rates of key vector-borne parasites [15,16–19]. Thus, it would be surprising if mosquito resistance mechanisms, for instance immune function, were not sensitive to changes in temperature.

Recently, we demonstrated in a malaria mosquito that expression of immune-related genes, together with functional measures of cellular and humoral resistance, do indeed vary with temperature [20]. Importantly, variation across temperature did not simply scale quantitatively with temperature; individual measures of immunity exhibited different thermal optima and distinct patterns of expression over time in response to different immune challenges [20]. This recent study, like others [12,21,22], considered a range of constant temperature environments. However, mosquitoes live in environments that often change dynamically throughout the day, between different habitats (e.g. indoors versus outdoors) and across seasons [14,23]. Evidence from numerous ectotherm systems (including mosquitoes) indicates that short-term temperature variation, for instance diurnal fluctuation, can affect a range of life-history traits relative to constant temperature environments [24,25–28]. In addition to extrinsic influences like temperature, the resistance of *Drosophila melanogaster* to bacterial infection has been shown to depend on the time of day flies are infected owing to a clock-regulated transient burst in the expression of innate immune genes [29]. Elements of mosquito behaviour [30–32], physiology [33,34] and some aspects of immune function [33] have also been shown to be under rhythmic control, suggesting that mosquitoes might vary in their susceptibility to infection with time of day. Whether diurnal temperature fluctuation and time of day of infection affect mosquito immunity and resistance remains largely unexplored.

In this study, we investigated how variation in mean temperature, diurnal temperature fluctuation and time of day shape the immunity and resistance of the Asian malaria vector *Anopheles stephensi*. We examined expression of three key immune-related genes (*defensin 1* (*DEF1*), *cecropin 1* (*CEC1*) and *nitric oxide synthase* (*NOS*)) and resistance to infection with *Escherichia coli. DEF1* and *CEC1* encode two antimicrobial peptides that are active against Gram-positive and -negative bacteria, filamentous fungi [35,36] and have been implicated to some extent with *Plasmodium* killing [37,38]. *NOS* encodes nitric oxide, an effector molecule that is involved in a multitude of immune responses toward a wide diversity of pathogens and parasites [39] and has been implicated as a major anti-malarial defence in the mosquito midgut epithelia [40,41]. Further, in a previous study, we have demonstrated that these three immune genes are differentially affected by changes in mean ambient temperature [20]. Building upon this research, we show direct effects of mean temperature, diurnal temperature fluctuation and time of day of infection, together with complex two- and three-way interactions, differentially affecting expression of individual immune genes, *in vivo* bacterial growth and mosquito survival. These results indicate that overall patterns of immunity and resistance are strongly influenced by environmental drivers and highlight the need to consider environmental context to better understand mosquito immunity and vector–parasite interactions.

## 2. Material and methods

### (a) Mosquito rearing and experimental design

We reared *An. stephensi* (Liston) under standard insectary conditions at 26 ± 0.5°C, 80% humidity and a 12 L : 12 D photo-period [20]. Upon adult emergence, males were separated from females and the males discarded. On day three post-emergence, female mosquitoes were then provided a bloodmeal from rats (Wistar, more than six weeks old). We conducted two experiments to assess how temperature, diurnal temperature fluctuation and time of day influence the expression of mosquito immune genes and resistance to bacterial infection. In both experiments, mosquitoes were anaesthetized on ice and challenged with 200 000 heat-killed (gene assay) or 2000 live (resistance assay) tetracycline-resistant GFP-expressing *E. coli* (dh5 alpha strain) through intrathoracic injection into the anepisternal cleft [42] with a mouth pipette and microcapillary glass needle. Mosquitoes were challenged/infected either in the morning (06.00) or evening (18.00) and were then distributed over 12 Percival incubators (three constant temperatures of 18°C, 26°C and 32°C; three diurnally fluctuating temperatures of 18 ± 6°C, 26 ± 6°C and 32 ± 6°C, and two replicates). All temperatures were controlled to ±0.5°C, with a relative humidity of 80 ± 5% (see the electronic supplementary material, methods).

Temperature fluctuation was programmed using an asymmetrical, minimum–maximum temperature model (see electronic supplementary material, methods, Parton–Logan model [43]), in which temperature follows a sinusoidal progression during the daytime and a decreasing exponential curve during the night. This temperature model reproduces realistic diurnal temperature fluctuations for a range of average temperatures [43,44]. This experimental design resulted in two levels of sampling times (06.00 and 18.00), three levels of constant ambient temperatures (18°C, 26°C and 32°C), two levels of fluctuation (constant versus variable) and two replicates (see electronic supplementary material, methods).

### (b) Immune challenge with *Escherichia coli*

For both experiments, we grew *E. coli* overnight in a Luria-Bertani's rich nutrient medium (LB broth) in a shaking incubator at 37°C, and a serial dilution was prepared from the overnight culture. The concentration of our bacterial stock was estimated by recording the absorbance (OD_600_) from each dilution using a Nanodrop (Thermal Scientific). We then either concentrated or diluted our stock to ensure a working concentration of 1 × 10^9^ *E. coli* per ml (i.e. 200 000 *E. coli* per injection) for the gene expression assays or 1 × 10^7^ *E. coli* per ml (i.e. 2000 *E. coli* per injection) for the mosquito resistance assay. To further confirm these estimates, we plated our injection solution in triplicate onto LB agar plates, placed them overnight into an incubator at 37°C, and counted the resulting colony forming units (CFUs) the next day. For the gene expression assays, we then killed the *E. coli* stock by autoclaving for 25 min. Heat-killed *E. coli* rather than live *E. coli* was used as our challenge in the gene expression assay in order to isolate the effects of experimental treatment on gene expression and to avoid temperature-mediated variation in bacterial growth within mosquitoes housed in different temperature treatments [20]. Further, we chose to work with a significantly higher dose of heat-killed *E. coli* and a lower dose live *E. coli* in the gene expression and mosquito resistance experiments, respectively, so as to maximize mosquito immune responses to immune challenge and to generate quantifiable estimates of bacterial growth across 24 h.

### (c) Gene expression assays: RNA collection, cDNA synthesis and quantitative PCR

Twenty-four hours post-challenge, mosquitoes were removed from their temperature treatment. To assess mortality, we first quantified and removed dead mosquitoes. Of the remaining mosquitoes, we killed them with chloroform and immediately stored them in RNA*later* RNA stabilization reagent at 4°C until termination of the experiment. Five mosquitoes from each treatment group (*n* = 360 total) were isolated individually in β-mercaptoethanol and RLT lysis buffer. Messenger RNA was extracted and quantified as described in [20]. Briefly, owing to the thermal sensitivity of *ribosomal protein S7* (*rpS7*) [20], a standard housekeeping gene in mosquito gene expression studies [41,45,46], we chose to quantify our diluted cDNA from our experimental samples by comparing their threshold cycle numbers against a standard curve generated from 1 : 10 serial dilutions of our standard sample (cDNA from a pool of four mosquitoes). We quantified cDNA counts for each gene of interest (*DEF1*, *CEC1*, *NOS* and *rpS7*) from individual mosquitoes collected across all experimental treatments relative to a standard curve produced for that gene. To account for individual differences in background gene expression, *rpS7* cDNA counts were included as a covariate in our statistical analyses (see below). Primers and probes were designed from *An. stephensi* and *Anopheles gambiae* sequences [20].

### (d) Measuring *in vivo* bacterial growth and mosquito mortality

To estimate mosquito resistance to infection, we recorded *in vivo* bacterial growth 24 h post-infection. To assess *in vivo* bacterial growth, live mosquitoes were put immediately in the freezer for 5 min. Once anaesthetized, mosquitoes were maintained on ice to limit any further bacterial growth prior to homogenization, and dead mosquitoes were counted and removed from each cup to assess mortality. From each experimental treatment group, three pools of three mosquitoes each were homogenized in 200 µl of 1× sterile phosphate-buffer saline (PBS) solution using a hand held mortar and pestle. After homogenization, we added 800 µl more of 1× PBS to each sample and made a series of dilutions in water (1 : 5, 1 : 10 and 1 : 50). We then plated 25 µl of each dilution in triplicate on tetracycline embedded, nutritive agar plates, which were then placed into an incubator at 37°C. Twenty-four hours later, we removed the plates and counted the number of colony forming units that had formed on each plate. Across both the gene expression and mosquito resistance assays, we recorded daily mosquito mortality.

### (e) Statistical analyses

All statistical analyses for these experiments were run in IBM SPSS Statistics v. 21.0 (IBM Corporation). Full factorial models from generalized linear model (GZLM) analysis were reduced through backward elimination of non-significant interactions. We assessed goodness of fit of the final models through model deviance, log likelihood values and model residuals. Covariates included in GZLMs were centred on their grand mean, and adjusted Bonferroni post hoc tests were used to identify significant pairwise comparisons. For all dependent variables analysed, we included the following factors in our model analysis: time of day (06.00 and 18.00), temperature (18°C, 26°C and 32°C), diurnal temperature fluctuation (±0°C and ±6°C) and replicate.

#### (i) Gene expression

To compare differences in average gene expression among our experimental treatment groups, we used the cDNA counts generated for each target gene from our standard curve analysis as our expression measure. Because the error structures for the expression of each gene were overdispersed, we transformed the cDNA counts (cube-root) for *DEF1*, *CEC1* and *NOS* and analysed all expression data with GZLMs assuming a normal distribution for the transformed dependent variables. Full factorial analyses were run for each gene separately to control for any differences in efficiencies among our assays as well as independence among our experimental samples. In addition to the factors described above, we included immune challenge (unmanipulated, injured or heat-killed *E. coli*) as an additional factor in all models. We also included the equivalently transformed and centred *rpS7* cDNA counts of each sample as a covariate in all models to adjust our estimated means of our target gene by any differences in baseline expression among mosquitoes and to improve overall model fit [20]. To assess mosquito mortality in response to heat-killed *E. coli* challenge, we used Poisson fit GZLM analysis (log link function) to compare how the average number of dead mosquitoes varied with the following fixed factors: temperature (18°C, 26°C or 32°C), diurnal temperature fluctuation (±0°C or ±6°C), time of day of immune challenge (06.00 or 18.00), immune challenge (unmanipulated, injured or heat-killed *E. coli*) and replicate.

#### (ii) Resistance to bacterial infection

To examine the effect of experimental treatment on bacterial growth within the mosquito, we ran a GZLM analysis assuming a gamma distribution (log link function) on the mean number of colony forming units. To assess mosquito mortality in response to live *E. coli* infection across 24 h, we used Poisson fit GZLM analysis (log link function) to compare how the average number of dead mosquitoes varied with the following fixed factors: temperature (18°C, 26°C or 32°C), diurnal temperature fluctuation (±0°C or ±6°C), time of day of infection (06.00 or 18.00) and replicate. We also incorporated centred mean *E. coli* growth (CFUs recovered) over 24 h as a covariate to account for any potential relationship between *in vivo* bacterial growth and mosquito mortality.

## 3. Results

### (a) *Defensin* expression

#### (i) Effects of immune challenge

**Figure 1.**
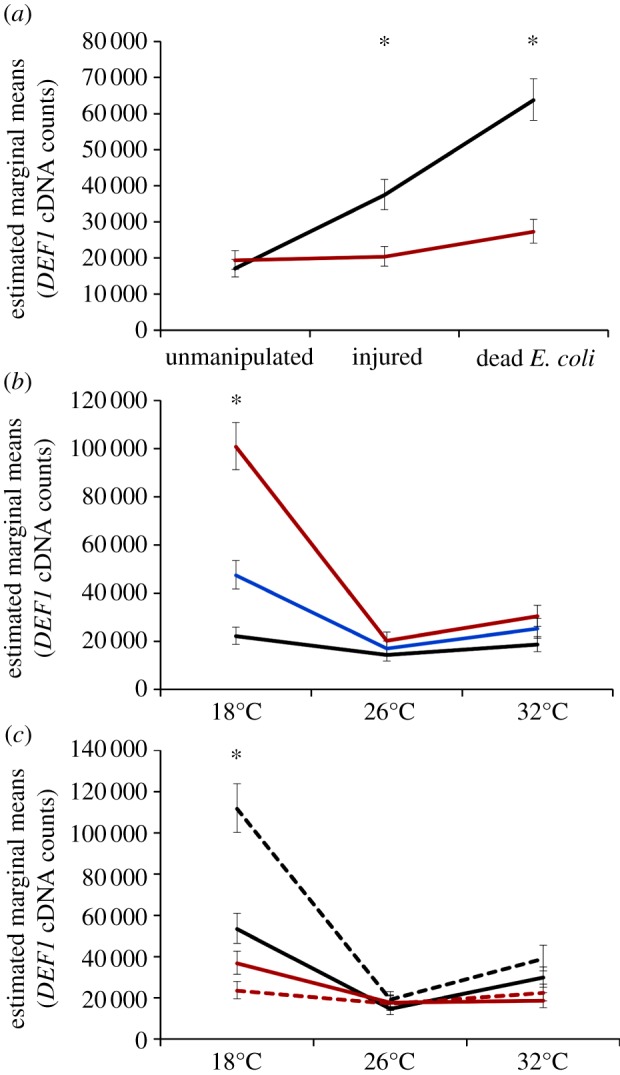
The effects of temperature, diurnal fluctuation and time of day on *defensin1* (*DEF1*) expression. (*a*) The expression of *DEF1* in response to immune challenge varies depending on the time of day mosquitoes are challenged (06.00, black line; 18.00, red line). (*b*) *DEF1* expression in response to immune challenge (unmanipulated, black line; injury, blue line; and heat-killed *E. coli*, red line) varies depending on the mean ambient temperature mosquitoes experience after immune challenge. (*c*) Independent of immune challenge, *DEF1* expression differs depending on the time of day (06.00, black line; 18.00, red line) a mosquito is challenged, the ambient temperature a mosquito is housed in, and whether or not there is diurnal temperature fluctuation (±0°C, solid lines; ±6°C, dashed lines). Asterisks denote significant differences of *p* < 0.05, and bars around population means represent standard errors.

Immune challenge significantly affected the expression of *DEF1* (table 1). As indicated by two, two-way interactions (time of day × immune challenge and temperature × immune challenge) the effect of immune challenge was mediated by the time of day the challenge was administered and variation in mean ambient temperature. For example, mosquitoes challenged with heat-killed *E. coli* in the morning expressed significantly more *DEF1* than injured (*p* < 0.0001) and unmanipulated mosquitoes (*p* < 0.0001; figure 1*a*). By contrast, there was no significant effect of injury or challenge with heat-killed *E. coli* in the evening. Mosquitoes challenged with heat-killed *E. coli* expressed significantly more *DEF1* than injured (*p* = 0.032) or unmanipulated mosquitoes (*p* < 0.0001), irrespective of time of day of immune challenge, when mosquitoes were placed into a cool temperature (18°C; figure 1*b*). Interestingly, *DEF1* expression levels did not significantly differ with immune challenge when mosquitoes were housed at warmer temperatures (26°C and 32°C; figure 1*b*).

**Table 1.** Final results from GZLM analysis of *DEF1*, *CEC1* and *NOS* expression. Significant effects for each gene are in italic (*p* < 0.05), and dashes indicate higher order interactions that were eliminated from the full model. (Omnibus tests confirmed that each fitted model was significantly different from its null model (*DEF1*: likelihood ratio 
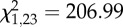
, *p* < 0.0001; *CEC1*: likelihood ratio 
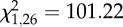
, *p* < 0.0001; *NOS1*: likelihood ratio 
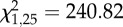
, *p* < 0.0001). Goodness of fit was assessed by evaluating potential overdispersion through model deviance scores and model residuals (*DEF1*: deviance value/d.f. = 7.12; *CEC1*: deviance value/d.f. = 2.65 and *NOS*: deviance value/d.f. = 1.71). For all three immune genes, a positive relationship existed with the expression of the housekeeping gene *rpS7* (Linear regression on model residuals: *DEF1*, *R*^2^ = 0.169, *F*_1,351_ = 10.33, *p* = 0.001; *CEC1*, *R*^2^ = 0.454, *F*_1,351_ = 91.0, *p* < 0.0001 and *NOS*, *R*^2^ = 0.726, *F*_1,351_ = 390.24, *p* < 0.0001).).

factors	*DEF1* (*n* = 353)			*CEC1* (*n* = 356)			*NOS* (*n* = 356)		
	Wald *χ*^2^	d.f.	*p*-value	Wald *χ*^2^	d.f.	*p*-value	Wald *χ*^2^	d.f.	*p*-value
intercept	*3901*.*05*	*1*	<*0*.*0001*	*4810*.*21*	*1*	<*0*.*0001*	*6221*.*50*	*1*	<*0*.*0001*
time of day	*34*.*06*	*1*	<*0*.*0001*	*8*.*51*	*1*	*0*.*004*	*75*.*11*	*1*	<*0*.*0001*
temperature	*95*.*21*	*2*	<*0*.*0001*	0.59	2	0.746	*51*.*98*	*2*	<*0*.*0001*
fluctuation	1.39	1	0.239	*6*.*53*	*1*	*0*.*011*	2.35	1	0.125
immune challenge	*62*.*55*	*2*	<*0*.*0001*	*14*.*80*	*2*	*0*.*001*	3.91	2	0.141
replicate	0.03	1	0.859	0.51	1	0.475	0.01	1	0.943
centred *rpS7* cDNA counts	*13*.*72*	*1*	<*0*.*0001*	*30*.*69*	*1*	<*0*.*0001*	*145*.*43*	*1*	<*0*.*0001*
time of day × temperature	*32*.*01*	*2*	<*0*.*0001*	*35*.*72*	*2*	<*0*.*0001*	5.22	2	0.074
time of day × fluctuation	*5*.*03*	*1*	*0*.*025*	0.25	1	0.681	0.24	1	0.623
time of day × immune challenge	*28*.*07*	*2*	<*0*.*0001*	5.03	2	0.081	1.99	2	0.370
temperature × fluctuation	0.18	2	0.913	*12*.*78*	*2*	*0*.*002*	1.37	2	0.503
temperature × immune challenge	*40*.*66*	*4*	<*0*.*0001*	*18*.*50*	*4*	*0*.*001*	7.53	4	0.110
fluctuation × immune challenge	2.17	2	0.338	5.57	2	0.062	0.56	2	0.754
time of day × temperature × fluctuation	*7*.*82*	*2*	*0*.*020*	*8*.*35*	*2*	*0*.*015*	—	—	—
time of day × temperature × immune challenge	—	—	—	*12*.*25*	*4*	*0*.*016*	*11*.*016*	*4*	*0*.*026*

#### (ii) Effects of environmental variation overall

Regardless of immune challenge, the expression of *DEF1* varied significantly with time of day, mean ambient temperature and diurnal temperature fluctuation as indicated by a three-way interaction (time of day × temperature × fluctuation; table 1). Mosquitoes placed into a diurnally fluctuating, cool environment in the morning expressed significantly more *DEF1* in general than mosquitoes housed in a constant, cool environment (06.00, 18 ± 0°C versus 18 ± 6°C, *p* = 0.001), or those mosquitoes placed into a constant (06.00, 18 ± 6°C versus 18.00, 18 ± 0°C) or fluctuating (06.00, 18 ± 6°C versus 18.00, 18 ± 6°C *p* < 0.0001), cool environment in the evening (figure 1*c*). This effect of diurnal fluctuation, however, disappears at warmer temperatures (26°C and 32°C) when mosquitoes were placed into their experimental treatments in the morning, and across all temperatures if mosquitoes were placed into their experimental treatments in the evening (figure 1*c*).

### (b) *Cecropin* expression

#### (i) Effects of immune challenge

**Figure 2.**
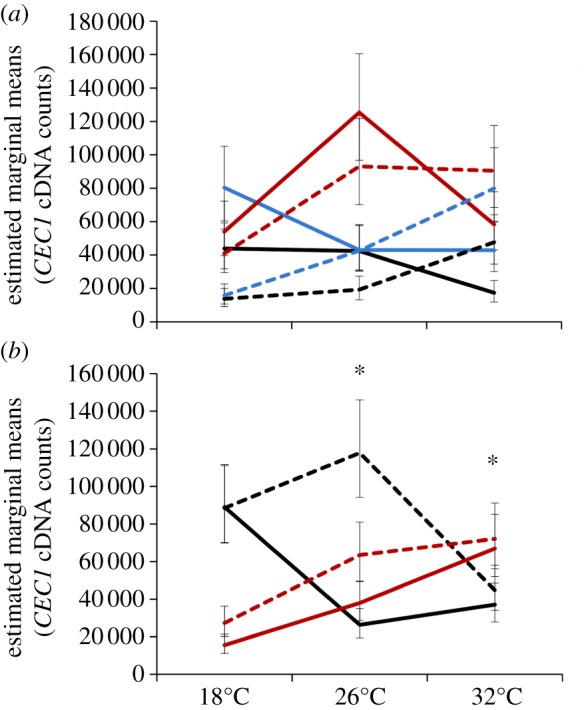
The effects of temperature, diurnal fluctuation and time of day on *cecropin1* (*CEC1*) expression. (*a*) The amount of *CEC1* in response to immune challenge (unmanipulated, black line; injury, blue line and heat-killed *E. coli*, red line) varied significantly depending on the average temperature mosquitoes experienced and the time of day challenge occurred (06.00, solid lines; 18.00, dashed lines). (*b*) Independent of immune challenge, the expression of *CEC1* varied significantly with the mean ambient temperature a mosquito experiences, the time of day mosquitoes were placed into their temperature treatments (06.00, black lines; 18.00, red lines), and whether or not there was diurnal fluctuation (±0°C, solid lines; ±6°C, dashed lines). Asterisks denote significant differences of *p* < 0.05, and bars around population means represent standard errors.

Like *DEF1* expression, *CEC1* expression was significantly affected by immune challenge (table 1). The effect of immune challenge on *CEC1* expression varied depending on the average temperature at which mosquitoes were housed and whether they were challenged in the morning or evening, as indicated by a significant two- (temperature × immune challenge) and three-way interaction (time of day × temperature × immune challenge; table 1). Mosquitoes expressed quantitatively and qualitatively different amounts of *CEC1* depending on the time of day of challenge, the mean ambient temperature mosquitoes were housed in and the nature of the immune challenge (injury versus heat-killed *E. coli*; figure 2*a*).

#### (ii) Effects of environmental variation overall

Like *DEF1*, *CEC1* expression was significantly affected by time of day, mean ambient temperature and diurnal temperature fluctuation regardless of immune challenge owing to two significant two-way interactions (time of day × temperature and temperature × fluctuation) and a significant three-way interaction (time of day × temperature × fluctuation; table 1). Mosquitoes placed into a 26°C, diurnally fluctuating environment, expressed significantly more *CEC1* than mosquitoes placed into a constant 26°C environment in the morning (*p* < 0.001; figure 2*b*); this occurs mainly because mosquitoes placed into constant environments in the morning express significantly less *CEC1* at 26°C relative to those placed into a constant, cool environment (06.00: 18 ± 0°C versus 26 ± 0°C, *p* = 0.009; figure 2*b*). By contrast, there is no effect of diurnal fluctuation when mosquitoes were placed into cooler (18°C) or warmer (32°C) environments in the morning or across all temperatures in the evening (figure 2*b*). Finally, mosquitoes placed into warm, constant environments in the evening expressed significantly more *CEC1* than those placed into cool, constant environments (18.00: 18 ± 0°C versus 32 ± 0°C; figure 2*b*).

### (c) Nitric oxide synthase expression

**Figure 3.**
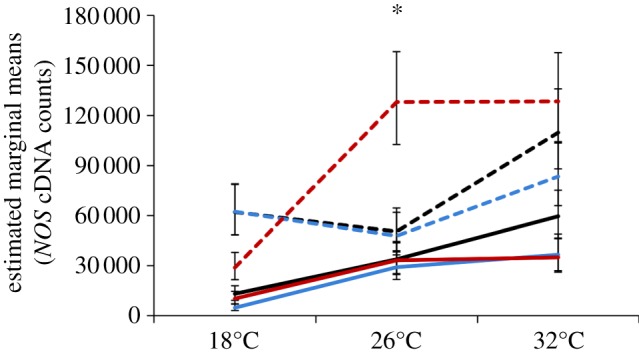
The effects of temperature, diurnal fluctuation and time of day on *NOS* expression. *NOS* expression in response to immune challenge (unmanipulated, black lines; injury, blue lines; and heat-killed *E. coli*, red lines) varied significantly in response to the mean ambient temperature mosquitoes experience and the time of day of immune challenge (06.00, solid lines; 18.00, dashed lines). Asterisks denote significant differences of *p* < 0.05, and bars around population means represent standard errors.

*NOS* expression was significantly shaped by immune challenge overall (table 1) and this effect was influenced by both the time of day of immune challenge and mean ambient temperature, illustrated by a significant three-way interaction (time of day × temperature × immune challenge; table 1). Mean ambient temperature only affects *NOS* expression in response to immune challenge when mosquitoes were challenged in the evening; mosquitoes challenged in the evening with heat-killed *E. coli* and placed into the standard rearing temperature for *An. stephensi* (26°C) expressed significantly more *NOS* than injured (*p* = 0.003) and unmanipulated mosquitoes (*p* = 0.003). In fact, immune challenge becomes irrelevant when mosquitoes were placed into cool (18°C) or warm (32°C) environments in the evening or if mosquitoes were challenged and housed at any temperature in the morning (figure 3). Finally, unlike *DEF1* and *CEC1* expression, diurnal temperature fluctuation did not significantly shape *NOS* expression, with mosquitoes expressing similar amounts of *NOS* in constant and diurnally fluctuating environments and across all mean ambient temperatures.

### (d) Mosquito resistance assays

#### (i) Bacterial growth

**Figure 4.**
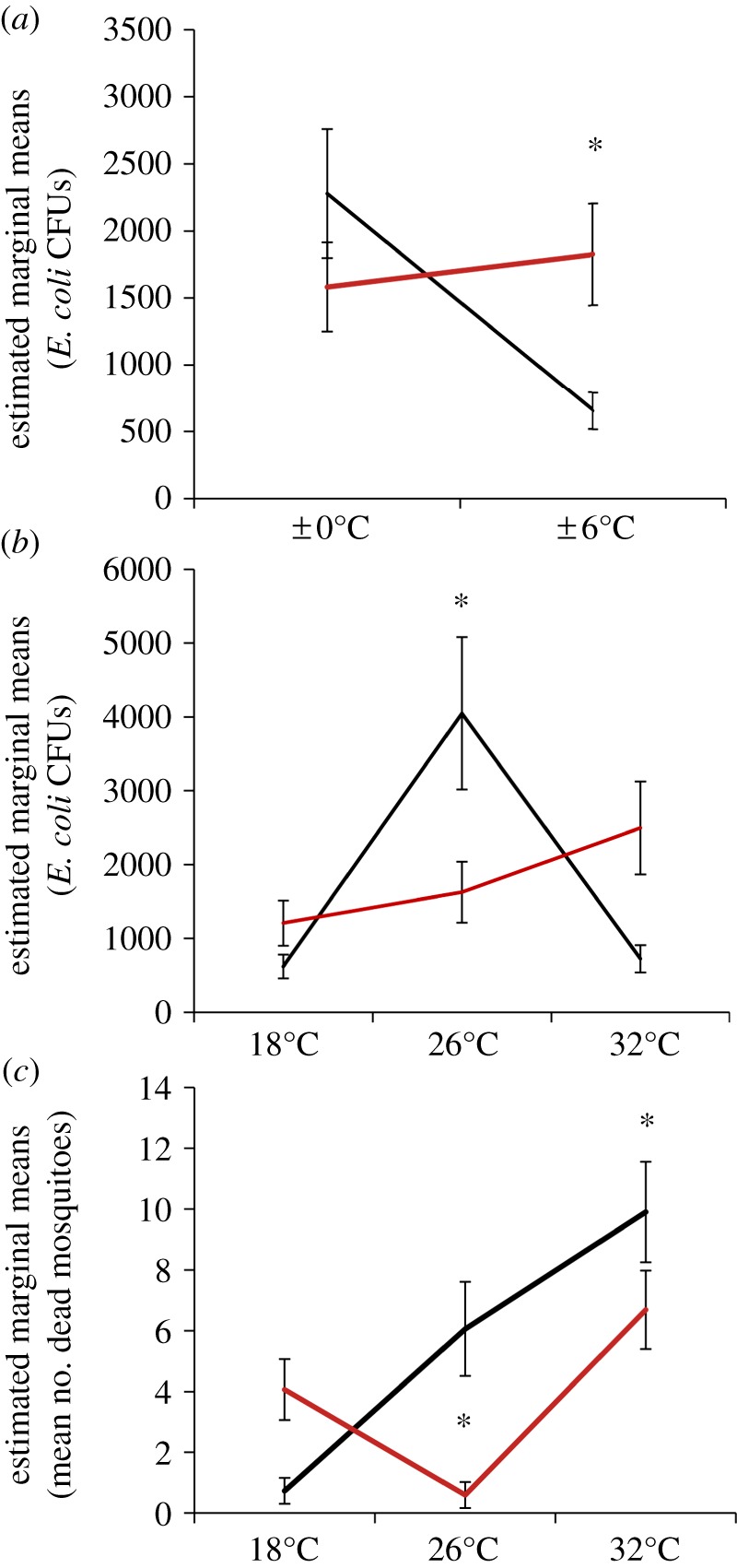
Bacterial growth *in vivo* and mosquito mortality are affected by ambient temperature, temperature fluctuation and time of day of infection. (*a*) Regardless of mean ambient temperature, bacterial growth (*E. coli* CFUs) within a mosquito was significantly affected by diurnal temperature fluctuation and time of day of infection (06.00, black line; 18.00, red line). The effect of mean ambient temperature on *E. coli* growth within the mosquito (*b*) and mosquito mortality (*c*) were also significantly shaped by the time of day of infection (06.00, black line; 18.00, red line). Asterisks denote significant differences (*p* < 0.05), and bars around population means represent standard errors.

*Escherichia coli* growth within the mosquito was significantly affected by changes in ambient temperature, diurnal temperature fluctuation and time of day at mosquitoes were infected (table 2), represented by two significant two-way interactions (time of day × fluctuation and time of day × temperature). Regardless of mean ambient temperature, mosquitoes significantly limited bacterial growth when they were infected in the morning and placed into a diurnally fluctuating environment relative to those infected in the morning and placed into a constant thermal environment (*p* = 0.008), or those infected in the evening and placed into a fluctuating environment (*p* = 0.019; figure 4*a*). The effect of diurnal temperature fluctuation no longer significantly affected mosquito resistance to bacterial growth when mosquitoes were infected in the evening (figure 4*a*). Overall, *in vivo* bacterial growth was greatest at 26°C when mosquitoes were infected in the morning relative to those also infected in the morning, but placed into a cool/warm ambient temperature (18°C versus 26°C, *p* = 0.016; 26°C versus 32°C, *p* = 0.020; figure 4*b*). However, when mosquitoes were infected with *E. coli* in the evening, the effect of ambient temperature on mosquito resistance is no longer significant (figure 4*b*).

**Table 2.** Final results from GZLM analysis for *E. coli* growth within the mosquito and mosquito mortality. Significant effects are in italic (*p* < 0.05) for each response variable, and dashes indicate higher order interactions that were eliminated from the full model. (Omnibus tests confirmed that each fitted GZLM model was significantly different from its null model (bacterial growth: likelihood ratio 
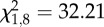
, *p* < 0.0001; mortality: likelihood ratio 
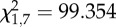
, *p* < 0.0001). Goodness of fit was assessed by evaluating potential overdispersion through model deviance scores and model residuals (bacterial growth: deviance/d.f. = 2.390; mortality: deviance/d.f. = 1.688).)

	bacterial survival (*n* = 72 pools)			mosquito mortality (*n* = 48)		
factors	Wald *χ*^2^	d.f.	*p*-value	Wald *χ*^2^	d.f.	*p*-value
intercept	*4978*.*56*	*1*	<*0*.*0001*	*136*.*37*	*1*	0.42
time of day	2.56	1	0.110	1.04	1	0.33
temperature	*17*.*97*	*2*	<*0*.*0001*	*42*.*26*	*2*	*<0.0001*
fluctuation	*6*.*57*	*1*	*0*.*010*	*5*.*76*	*1*	0.068
replicate	0.05	1	0.827	*13*.*32*	*1*	*0.001*
centred mean *E. coli* CFUs	—	—	—	0.26	1	0.607
time of day × temperature	*19*.*32*	*2*	<*0*.*0001*	*17*.*45*	*2*	*<0.0001*
time of day × fluctuation	*10*.*67*	*1*	*0*.*001*	—	—	—

#### (ii) Mosquito mortality

Patterns of mosquito mortality were similar across both the gene expression (see electronic supplementary material, results) and mosquito resistance assays, with significant effects of mean ambient temperature and an interaction between time of day and ambient temperature (time of day × temperature; table 2 and electronic supplementary material, results). Overall, the mean number of dead mosquitoes was significantly higher (three to four times higher) when mosquitoes were infected with live *E. coli* compared with dead *E. coli* (compare results in electronic supplementary material, figure SI 1 with figure 4). Further, *in vivo* bacterial growth was not a significant predictor of mosquito mortality in the resistance assay (table 2). In both the gene expression and mosquito resistance assays, the number of dead mosquitoes overall increased when mosquitoes were infected in the morning and then were housed at a warm temperature (32°C) relative to cooler temperatures (both assays: 18°C versus 26°C, *p* < 0.0001; 18°C versus 32°C, *p* < 0.0001). This trend, however, qualitatively changes in both assays when mosquitoes are infected in the evening. In the gene expression assay, there was no significant effect of temperature on mosquito mortality when mosquitoes were infected in the evening (see electronic supplementary material, results), whereas in the mosquito resistance assay mosquitoes infected in the evening experienced significantly more mortality when placed into cool (18°C) and warm (32°C) temperatures (18°C versus 26°C, *p* = 0.0016; 26°C versus 32°C, *p* < 0.0001; figure 4*c*). We did have a significant replicate effect in the resistance assay, with mosquitoes in replicate two experiencing higher mortality on average than those in replicate one (table 2); however, the effects of mean ambient temperature, diurnal temperature fluctuation and time of day did not qualitatively differ between replicates.

## 4. Discussion

Our results add to a growing body of literature demonstrating the potential for complex interactions among environmental variables and invertebrate immunity/resistance [28,47,48]. Both the expression of mosquito immune genes (*DEF1*, *CEC1* and *NOS*) and mosquito resistance to bacterial infection (i.e. *in vivo* bacterial growth and mosquito mortality) were strongly shaped by realistic environmental variation. Such effects are likely to contribute to marked heterogeneity in immune function and resistance (including natural refractoriness to malaria parasites) across time and space, and challenge the robustness of the mechanistic insights gained from studies conducted under a constrained set of laboratory conditions.

Our constant and fluctuating temperature treatments were set up to provide approximately equivalent mean temperatures to one another. However, because we used a realistic asymmetric diurnal fluctuation rather than a symmetrical sine function, the cumulative degree hours were not identical between the paired temperatures, and the actual daily mean temperatures in the fluctuating treatments exceeded those of the constant treatments by around 0.1°C. Such small differences are unlikely to explain the magnitude of the effects of fluctuation on our response variables. Moreover, we observed significant interactions between diurnal temperature fluctuation and time of day, suggesting that the portion of the diurnal fluctuation (e.g. cooling versus warming) experienced directly following challenge/infection is important.

Unsurprisingly, immune challenge significantly increased the expression of antimicrobial peptides and *NOS*; in all cases, this effect was moderated by mean ambient temperature, diurnal temperature fluctuation and the time of day of immune challenge. Mean ambient temperature differentially shaped the expression of mosquito immune genes in response to heat-killed *E. coli*, with *DEF1*, *CEC1* and *NOS* expression experiencing diverse thermal maxima, which replicates well with our previous study [20]. The expression of both antimicrobial peptides and *NOS* were also significantly affected by the time of day mosquitoes were immune-challenged, with time of day affecting the precise direction of antimicrobial peptide and *NOS* expression in different ways (i.e. antimicrobial peptides and *NOS* were expressed more, on average, when challenge occurred in the morning and evening, respectively). These effects occurred whether or not mosquitoes were in a constant or fluctuating thermal environment suggesting that *DEF1*, *CEC1* and *NOS* are under some sort of rhythmic control. This seems reasonable considering a significant portion of the *An. gambiae* genome exhibits diel rhythms in expression [33], and *D. melanogaster* has a number of clock-regulated immune genes [29]. Yet, there were also interactions between temperature fluctuation and the time of day suggesting that rhythmic effects can integrate with diurnal temperature profiles to influence immunity and resistance. The mechanisms are currently unclear, but the interaction could occur for a variety of reasons. First, immune molecules could interact with, or compete for resources with other metabolic processes resulting in a redistribution of immune molecules based on an organism's activity schedule [49–51] and with physiological processes required for temperature acclimation. This mechanism could also be driving the effect of the three-way interaction among mean ambient temperature, diurnal fluctuation and time of day on gene expression of antimicrobial peptides independent of immune challenge. Many studies have demonstrated circadian and seasonal changes in physiology and immunity in a wide diversity of organisms [52–54], suggesting that mosquito susceptibility could change with particular aspects of a mosquito's life history. Second, immune responses and parasite processes could have different thermal optima [20,55]; thus, the temperature at and immediately following infection could dramatically impact parasite establishment [11,41,56]. Or third, as suggested by the observed interactions among temperature, diurnal temperature fluctuation and immune challenge, could be some combination of both.

We initially expected bacterial growth within the mosquito, as well as mosquito mortality, to increase with temperature regardless of time of infection because *E. coli* is typically cultured at 37°C. However, this was not the case and *in vivo* bacterial growth was not a significant predictor of mosquito mortality. Alternatively, owing to the qualitatively similar trends in mosquito mortality in the gene expression assays, the mosquito mortality in the resistance assay could be owing to increased immunopathology associated with live infections.

There are some suggestive associations between *in vivo* bacterial growth, mosquito mortality and the patterns of antimicrobial peptide expression we observe. *E. coli* experienced increased within host growth when mosquitoes were infected in the evening and subjected to constant temperature environments, whereas mosquitoes in general expressed the least *DEF1* and *CEC1* in these environments in response to heat-killed *E. coli* (figures 1 and 2). Thus, lower levels of *DEF1* and *CEC1* may indicate lower immune responses to infection under these circumstances. There might also be a link between bacterial growth and *NOS* expression because NOS is a key enzyme secreted by mosquito midgut, fat body and haemocytes in the defence against bacterial pathogens [42]. If NOS enzyme production correlates with *NOS* expression, significant increases in *NOS* expression in mosquitoes challenged with heat-killed *E. coli* in the evening might explain why bacterial growth and mosquito mortality was highest on average when mosquitoes were challenged in the morning.

However, the functional role of these immune measures remains slightly uncertain. For example, there is some evidence for differential activity of antimicrobial peptides *in vitro* as compared with *in vivo* [57]. Moreover, the expression of antimicrobial peptides and *NOS* interact with other components of the immune response mosquitoes mount toward bacterial infection. Both antimicrobial peptides and the NOS enzyme have been shown to interact with a thioester-containing protein (*TEP1*), a complement-like protein, which functions as an opsonin that binds covalently to the surface of both Gram-positive and -negative bacteria stimulating their clearance through phagocytosis by circulating granulocytes [58,59]. In the absence of gene silencing, the mechanistic link between our immune measures and resistance is currently unclear, and we cannot ultimately say whether these responses are resistance mechanisms or a consequence of infection [60]. What these results definitively demonstrate is how environmental variation can influence mosquito immunity and resistance in qualitatively diverse ways that could be biologically important and are both complex and nonlinear.

In this study, we considered only three immune-related genes and one pathogen; however, there is little reason to assume these to be uniquely sensitive to environmental variation. The effects we observe might be especially important for pathogens and parasites with developmental stages that are sensitive to both temperature and timing of innate immune responses, like malaria [19,41,61]. Our data suggest that mean temperature, diurnal temperature variation and the timing of blood feeding could combine to determine net vector competence [28,62,63]. Such effects are also likely to influence the efficacy of prospective vector control tools that exploit immune mechanisms or use pathogens or parasites [45,64,65], and might even affect conventional tools, for instance chemical insecticides. Indeed, insecticide resistance has been shown to vary in *Aedes aegypti* depending on time of day of exposure [66,67] and to vary with temperature in *An. stephensi* [68].

In summary, altering just one variable (e.g. time of day or temperature variation) can dramatically affect patterns of gene expression and resistance. In reality, we expect multiple interacting variables to change simultaneously. With such complexity, figuring out how environmental parameters mechanistically influence mosquito physiology, immunity and resistance is non-trivial. Nonetheless, our results clearly highlight the need to consider mosquito immunity and resistance beyond the limits of standard insectary conditions.

## Data accessibility

Data available from the dryad digital repository: http://dx.doi.org/10.5061/dryad.
